# The Challenging Scenario of Cancer Treatment for People with HIV: Clinical Experience with Immune Checkpoint Inhibitors

**DOI:** 10.3390/curroncol32030164

**Published:** 2025-03-13

**Authors:** Tindara Franchina, Patrizia Carroccio, Ylenia Russotto, Mariapia Marafioti, Paola Muscolino, Francesco Monaco, Antonio Bottari, Silvana Parisi, Giovanni Francesco Pellicanò, Massimiliano Berretta

**Affiliations:** 1 Medical Oncology Unit, Department of Human Pathology “G. Barresi”, University of Messina, 98125 Messina, Italy; tindara.franchina@unime.it (T.F.); patrizia.carroccio@studenti.unime.it (P.C.); mariapia.marafioti@studenti.unime.it (M.M.); paola.muscolino@studenti.unime.it (P.M.); 2 Unit of Infectious Diseases, Department of Clinical and Experimental Medicine, University of Messina, 98125 Messina, Italy; ylenia.russotto@studenti.unime.it (Y.R.); giovanni.pellicano@unime.it (G.F.P.); 3 Section of Thoracic Surgery, Department of Biomedical, Dental, Morphological and Functional Imaging Sciences, University of Messina, 98125 Messina, Italy; francesco.monaco@unime.it; 4 Section of Radiological Sciences, Department of Biomedical, Dental, Morphological and Functional Imaging Sciences, University of Messina, 98125 Messina, Italy; bottaria@unime.it; 5 Radiation Oncology Unit, Department of Biomedical, Dental and Morphological and Functional Imaging Sciences, University of Messina, 98125 Messina, Italy; silvana.parisi@polime.it

**Keywords:** people with HIV, HIV and cancer, acquired immune deficiency syndrome, cancer treatments, immune checkpoints inhibitors, cancer immunotherapy

## Abstract

Over the past decade, there has been a notable increase in the utilization of immune checkpoint inhibitors in cancer care, transforming the therapeutic landscape for several types of solid tumors. This development has not only expanded the indications for treatment but has also significantly influenced management strategies and prognostic outcomes for specific subsets of cancer patients. In contrast to the general population of cancer patients, individuals diagnosed with both HIV and cancer encounter significant differences in treatment approaches and outcomes. Consequently, this population demonstrates a significantly increased rate of specific mortality for several common types of cancer. Recent studies have reported significant insights into the use of immune checkpoint inhibitors among this patient group. However, the data remain insufficient, and there are still recognized barriers and limitations regarding the use of these agents in cancer patients. Real-world data and reports from clinical practice offer critical perspectives, enabling the sharing of clinical experiences and assisting in navigating complex management decisions. This report outlines two cases of patients with concurrent HIV and cancer who were administered ICIs in diverse clinical settings, highlighting the necessity of cooperation between oncologists and HIV specialists to provide patients with cutting-edge and increasingly tailored treatment options.

## 1. Introduction

The extensive implementation of antiretroviral therapy (ART) has resulted in a significant reduction in the incidence of opportunistic infections associated with HIV. However, cancers have become the leading cause of mortality among people living with HIV (PLWH) in many developed countries [1].

It is widely established that PLWH face an elevated risk of not only developing acquired immune deficiency syndrome (AIDS)-defining cancers (i.e., Kaposi’s sarcoma, non-Hodgkin lymphoma, cervical cancer, and anal cancer) [2] but also experience a rise in incidence and mortality for a wide spectrum of non-AIDS-defining cancers (NADCs) during their lifetime [3].

Therefore, there is an urgent need to raise awareness of risk factors, implement primary prevention initiatives, and develop early and adaptive cancer screening and effective treatment strategies.

Compared with the general cancer population, patients diagnosed with both HIV and cancer experience significant treatment and outcome disparities and, consequently, have a substantially higher rate of specific mortality for many common cancer types [4].

This disparity in survival is influenced by multiple factors, including an advanced stage at the time of diagnosis, more aggressive cancer phenotypes due to immune dysregulation and immunosuppression, decreased efficacy or increased toxicity associated with cancer treatments, and the impact of other comorbidities.

Over the past decade, the use of immune checkpoint inhibitors (ICIs) in oncology has grown significantly, changing the treatment paradigm for multiple types of malignant neoplasms [5]. This advancement has expanded treatment indications and significantly impacted management strategies and prognostic outcomes in subsets of cancer patients [6]. Historically, HIV patients with cancer were systematically excluded from cancer ICI trials.

Recent studies have presented intriguing data on using ICIs in this population [7].

However, available data remain limited, and obstacles and limitations in the use of these drugs in cancer patients are still documented.

Real-world data and clinical practice reports provide valuable insights, the opportunity to share clinical experiences, and support for challenging management decisions.

In this report, we present two cases of patients with concurrent HIV and cancer treated with ICIs in different clinical settings, supported by a team-oriented approach involving both oncologists and HIV specialists, encouraging the use of immunotherapy in this setting.

## 2. Case Description

We report two cases of HIV-positive patients undergoing immunotherapy treatment for cancer. Informed consent was obtained from both patients involved in this report.

The first case involved a 65-year-old female patient with a history of tobacco abuse and a diagnosis of HIV in 1991, untreated in the last two years. Her relevant medical history included a previous hepatitis C infection, gallstones, and a major stroke resulting in permanent hemiplegia. Furthermore, she had additional comorbidities, including a hiatal hernia, hypertension, osteoporosis, and an anxious–depressive syndrome.

Routine radiography revealed a nodular shadow in the left lung upper field, whereas a chest computed tomography (CT) showed a mass in the apico-dorsal segment of the left upper lobe measuring 15 mm in diameter and bilateral multiple variable-size lung nodules with a maximum diameter ≤8 mm. Lymphadenopathies were reported in the paratracheal and subcarinal region and aorto-pulmonary window.

After a multidisciplinary assessment, the staging was completed with a PET/CT scan, confirming a stage IVA disease and a CT-guided biopsy of the nodule was performed.

Pathomorphological examination led to the identification of poorly differentiated adenocarcinoma with a low PDL1 expression (TPS 1–49%).

The subsequent molecular characterization revealed the presence of KRAS-G12C mutation.

Because of her HIV-positive status, a pretreatment assessment at the Infectious Diseases Unit of our hospital was performed.

The patient reported that she underwent follow-up care at another hospital; however, she had not been consistently receiving antiretroviral therapy as part of her treatment plan. Upon evaluation, her HIV-RNA load was found to be 110,000 IU/mL. Consequently, the medical team initiated an antiretroviral therapy regimen comprising a combination of bictegravir, emtricitabine, and tenofovir alafenamide. The planning of first-line cancer treatment was effectively coordinated by oncologists and specialists in infectious diseases to evaluate drug interactions and personalize therapeutic approaches using the proactive management of toxicities.

The pretreatment viral load and lymphocyte counts were, respectively, as follows: HIV-RNA flare, 72 IU/mL; absolute lymphocyte count, 1512/mmc; CD3, 86.3% (1305/mmc); CD4, 18.6% (281/mmc); CD8, 64.2% (971/mmc); and CD4/CD8, 0.29%. After conducting a comprehensive re-evaluation that included serological and virological assessments due to her HIV-positive status, the patient was suitable for starting a chemo-immunotherapy treatment based on tumor histology, the advanced stage of the disease, which ruled out locoregional treatment options, the expression of PD-L1, and the results of the molecular analysis.

The treatment was started by administering the chemotherapy regimen of carboplatin plus pemetrexed reduced by 20%, and without the addition of immunotherapy, to evaluate safety and tolerability due to the frailty status associated with the comorbidity burden, including permanent hemiplegia and the concomitant HIV status, which results in a poor hematologic reserve.

Additionally, the concurrent HAART therapy may exacerbate hepatic and renal toxicity.

After administering the first cycle of chemotherapy and confirming good tolerability, concomitant chemo-immunotherapy with a fixed dose of Pembrolizumab (200 mg) was started at the second cycle.

This strategy is crucial given the palliative aim of first-line systemic treatment to achieve an appropriate response while minimizing toxicity.

The patient completed three cycles of chemo-immunotherapy and then started maintenance therapy with pemetrexed and Pembrolizumab. A tumor assessment with a CT scan after six months of treatment showed a partial response with a relevant reduction in the apico-dorsal segment of the left upper lobe.

At the time of this report, the patient is continuing her antiretroviral therapy and undergoing periodic evaluation of the viral load and lymphocyte count. The most recent assessment indicated that the HIV-RNA load is undetectable, and the lymphocyte count is as follows: absolute lymphocyte count, 1430/mmc; CD3, 77.7% (1111/mmc); CD4, 25% (357/mmc); CD8, 51% (729/mmc); and CD4/CD8, 0.49%.

**Figure 1:**
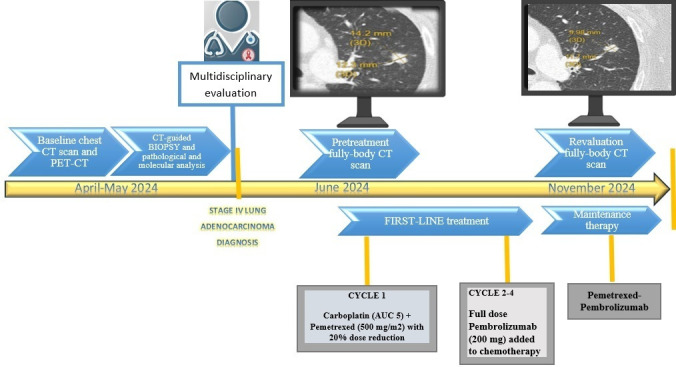
Overview of the diagnostic and therapeutic journey experienced by the patient diagnosed with metastatic lung adenocarcinoma and HIV treated with a chemo-immunotherapy combination. Overall, the treatment was well tolerated, with only one reported episode of G2 neutropenia and an increase in transaminases occurring after the second cycle of therapy, which were effectively managed with supportive care and a 7-day intercycle delay.

An outline of the diagnostic and therapeutic process is presented in Figure 1.

The second case involved a 60-year-old male diagnosed with squamous cell carcinoma of the scalp who had been HIV-positive since 1994. Initially, he received ART with Azidothymidine, and in 1997, he transitioned to a triple combination therapy. However, drug resistance developed due to inadequate adherence to the treatment regimen. Currently, the patient is being treated with bictegravir/emtricitabine/tenofovir alafenamide and is under infectious disease unit follow-up at a different medical facility.

His medical history also includes recent hepatitis B virus (HBV) positivity, monitored through hepatological follow-up, as well as multiple occurrences of squamous cell and basal cell carcinomas.

In November 2023, the patient was diagnosed with G3 squamous cell carcinoma localized in the left frontal region and was subsequently treated surgically.

The tumor infiltrated the full-thickness dermis and extended into the hypodermis.

After receiving adjuvant radiotherapy, a local recurrence was observed within a few months. In June 2024, a PET-CT scan was performed to complete the disease staging and indicated the presence of two adjacent areas with significant and uneven tracer uptake in the left frontal area, extending to the skull plate, which was partially eroded. As the disease had progressed to a locally advanced stage and was not suitable for locoregional treatment, the administration of immunotherapy with cemiplimab was recommended.

To facilitate the initiation of systemic treatment, a pretreatment assessment was performed with the HIV specialist, and drug interactions and potential toxicities were evaluated.

The pretreatment lymphocyte count was absolute lymphocyte count, 3690/mmc; CD3, 91% (3358/mmc); CD4, 5% (185/mmc); CD8, 85% (3137/mmc); and CD4/CD8, 0.06%.

Consequently, the patient started immunotherapy with cemiplimab.

**Figure 2:**
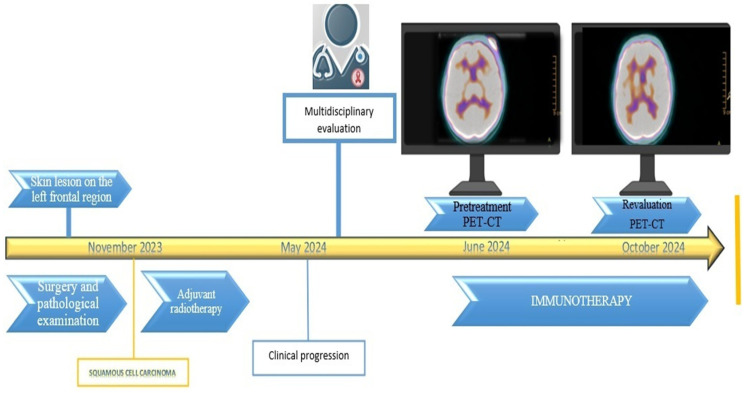
Overview of the diagnostic and therapeutic journey experienced by the patient diagnosed with locally advanced skin carcinoma of the left frontal region and HIV, treated with immunotherapy.

An outline of the diagnostic and therapeutic process is summarized in Figure 2.

**Figure 3:**
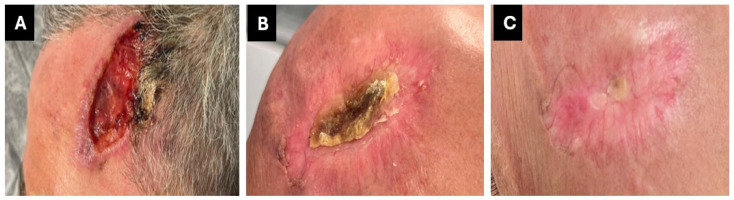
(**A**) Pretreatment clinical evaluation; (**B**) clinical evaluation after three months of cemiplimab treatment; and (**C**) clinical evaluation after six months of immunotherapy.

A clinical and PET scan re-evaluation in October 2024 revealed a complete response (Figure 3). Immunotherapy was well tolerated and did not interact with the ongoing HIV treatment. These factors, along with the significant efficacy of the drug that resulted in a complete response, have certainly improved the patient’s quality of life and psychological well-being.

At the time of this report, the patient shows adequate viro-immunological compensation (absolute lymphocyte count 1515/mmc; CD3 75%, 1136/mmc; CD4 33%, 500/mmc;) and continues immunotherapy with clinical and radiological monitoring and antiretroviral therapy, undergoing periodic evaluation with HIV specialists.

## 3. Discussion

The introduction of highly active ART has significantly changed the treatment landscape for PLWH, leading to the expectation that many will achieve life expectancy comparable to that of the general population [7].

The management of both HIV and cancer presents significant complexities, necessitating a collaborative approach between oncologists and HIV specialists. Adjustments to ART may be required to minimize potential interactions between cancer therapies and HIV treatments.

Cancer therapy often involves elaborate treatment strategies that incorporate the use of multiple drugs or concurrent locoregional and systemic approaches to achieve optimal patient outcomes.

Experts in several fields collaborate with oncologists to tackle the distinct challenges encountered by cancer patients, who are often more susceptible to infectious complications because of their primary illness and the immunosuppressive nature of cancer treatments.

In cancer patients with HIV, a critical aspect is a comprehensive evaluation of the safety and possible interactions of the drugs administered in cancer treatment to develop proactive strategies to reduce toxicities and the risk of developing infections [8].

Although cytotoxic agents may exhibit potential immunosuppressive effects and increase susceptibility to infections by directly reducing the immune response or disrupting the body’s natural protective barriers against pathogens, some chemotherapy drugs may directly stimulate antitumor immunity and enhance immunotherapy outcomes [9].

Several studies have shown that the use of ICIs in PLWH who are diagnosed with advanced cancer is both feasible and safe. The evidence indicates that these treatments are well tolerated, with no notable unexpected toxicities or viral reactivation occurring within this patient population [7].

Although patients with HIV have been excluded from cancer ICI trials [10], ongoing clinical trials and real-world studies, particularly from the CATCH trial [11], indicate a promising role for immunotherapy in treating this population.

However, the contribution of individual clinical experiences and case reports remain essential for a more comprehensive exploration of this therapeutic option for these patients, given the heterogeneity of their clinical presentations. Developing specific clinical pathways or sharing outpatient facilities to effectively manage these patients would be highly beneficial. Establishing dedicated databases and sharing clinical experiences could significantly enhance patient management in clinical research.

The simultaneous use of HAART and ICIs raises concerns related to pharmacokinetic interactions, the risk of inflammatory immune reconstruction syndrome, and the overall impact on the activity and safety of treatment in these patients [12].

Since HAART itself can lead to immune reconstitution syndrome, the administration of ICIs can further amplify this risk by inciting a solid immune response that could cause inflammatory reactions not only in cancer sites but also in otherwise healthy tissues.

In addition, the current data on antiretroviral therapy–ICI interactions are limited by the heterogeneity of study populations and the variability of therapeutic approaches.

Therefore, there is a critical need for increased vigilance in monitoring patients undergoing HAART and ICIs, given that the risks associated with autoimmune reactions and infectious complications necessitate the implementation of proactive management strategies. Recent studies have revealed new insights indicating that combinations of antiretroviral therapy can have varying impacts on the gut microbiome, immune activation, and the process of microbial translocation [13].

These data are highly significant in patients undergoing treatment with ICIs, as the gut microbiome may play a role in modulating the response to immunotherapy [14].

There is a notable lack of data on potential interactions between antiretroviral drugs and ICIs, depending on the pharmacokinetic properties. It is imperative to be aware of these interactions, encourage collaborative care, and implement standardized treatment guidelines [12].

Additionally, an essential aspect is the education of patients with HIV regarding cancer prevention, alongside the training of upcoming oncologists and infectious disease experts to develop collaborative frameworks.

This approach aims to bridge gaps in treatment and care, ensuring that HIV-positive cancer patients can benefit from the latest innovative therapies following international guidelines.

The clinical cases presented in this article highlight several significant aspects.

In each case, we administered the standard oncological treatment recommended by national and international guidelines, considering the histological diagnosis and the stage of the disease.

The involvement of a multidisciplinary team ensures that the HIV status does not pose a barrier to providing patients with appropriate treatment, thereby ensuring the best therapeutic opportunities. In the first case, a shared management strategy enabled the effective treatment of a frail patient with several comorbidities who experienced positive outcomes from immunotherapy, which was well tolerated.

Reducing the first cycle of chemotherapy, conducted without immunotherapy, was a vital strategy for evaluating the patient’s compliance and tolerability.

This approach facilitated the continuation of chemotherapy while integrating standard-dose immunotherapy, thus providing the optimal therapeutic option for this setting. Therefore, considering the patient’s clinical conditions and metastatic stage, improving clinical outcomes and ensuring a satisfactory quality of life was essential.

Moreover, it is crucial to highlight that the patient presented a KRAS G12C mutation, which is recognized to substantially influence the efficacy of PD-1/PD-L1 pathway-related inhibitory therapy, resulting in notable effects on progression-free survival (PFS) and overall survival (OS).

The majority of KRAS G12C mutations result from smoking and are correlated with an elevated tumor mutational burden, which is indicative of the potential response to immune checkpoint inhibitors [15]. Research indicates that aberrant proteins produced by mutant RAS can trigger immune responses, emphasizing the promise of immunotherapy for addressing RAS-mutant cancers.

Therefore, the opportunity to receive chemo-immunotherapy has allowed this patient to benefit from the optimal treatment option, considering both the stage of the disease and the tumor molecular profiling. This tailored approach was supported by a multidisciplinary team that involved oncologists and HIV specialists.

The second case emphasizes the role of cemiplimab in a patient with HIV and immune system dysfunction, highlighting the intricate challenges faced by individuals with both HIV and cancer and the need for additional data to optimize their management.

Immune-related adverse events (irAEs) were not observed in either of the patients.

The management of individuals diagnosed with both HIV and cancer requires a complex strategy that highlights the critical role of multidisciplinary teamwork and a holistic, patient-centered approach to care. With the growing population of cancer patients who are also HIV-positive, healthcare providers face specific challenges to achieve optimal health outcomes.

Central to effective multidisciplinary collaboration is open communication. Regular case discussions between experts can ensure that all team members are informed about patient treatment and the evaluation of clinical conditions and allow timely interventions when complications arise. In addition, involving patients in discussions about their options and treatment preferences can improve their satisfaction and promote adherence to prescribed therapies.

Psychologists contribute to mental health needs, which are usually exacerbated in patients dealing with the double diagnosis of HIV and cancer, especially in primary care environments.

Training and education among health service providers on the management of HIV patients and cancer patients are fundamental. Continuous professional development programs can promote an understanding of the complex interactions between HIV and cancer treatments to make informed choices that optimize results.

The implementation of palliative care strategies at the beginning of the treatment process for HIV and cancer patients can significantly improve their quality of life.

## 4. Conclusions

The significant positive results observed in these patients highlight the essential role of a multidisciplinary approach and the collaborative work between oncologists and HIV specialists.

Research and clinical reports indicate that checkpoint immunotherapy is safe and effective for HIV-positive people with cancer. To address disparities in clinical settings and deliver equitable care to every patient, the role of teamwork is essential.

The main aim of our report is to promote adherence to guidelines for implementing appropriate therapeutic strategies in this patient population, ensuring optimal therapeutic responses. While the limited number of cases and the short follow-up period, given that treatments are still ongoing, restrict clinical evaluations, the most significant finding from our study is the critical role of a multidisciplinary team in clinical practice within this setting.

Despite the complexities of the clinical and oncological landscape, along with the concurrent presence of HIV, the management of these patients can be optimized with dedicated support from oncologists and infectious disease specialists, especially in the absence of definitive data regarding pharmacological interactions, tolerability profiles, and specific clinical benefits.

To respond to these limitations, multicenter prospective studies will be essential to define clinical indications that can optimize the safety and efficacy of combined approaches in this unique patient cohort.

Understanding the interaction between HIV status and cancer immunotherapy is crucial, as these factors can significantly influence treatment outcomes. Biomarker development represents another critical area of future research to improve the results of immunotherapy for HIV cancer patients. The identification of predictive biomarkers and prognosis can facilitate patient stratification and allow personalized treatment approaches.

An intriguing area of research is exploring the tumor microenvironment in patients with HIV, which may uncover unique mechanisms of tumor immune evasion. Identifying these mechanisms could lead to enhancements in the efficacy of current therapeutic options.

In conclusion, the intersection of HIV and cancer treatment reveals a complex landscape that requires comprehensive investigation. It is crucial to bridge the current knowledge gaps by conducting larger cohort studies and the exploration of novel biomarkers to improve treatment efficacy for this vulnerable group of patients. Promoting collaborative and multidisciplinary efforts will be essential for deepening our understanding and optimizing therapeutic strategies. Future research should concentrate on the critical areas to ensure that cancer patients living with HIV can access the benefits of immunotherapy equitably.

## Data Availability

The original data presented in this study are included in the article. Further inquiries can be directed to the corresponding author.

## Abbreviations

The following abbreviations are used in this manuscript:

AIDS	Acquired immune deficiency syndrome
ART	Antiretroviral therapy
CT	Computed tomography
HAART	Highly active antiretroviral therapy
ICIs	Immune checkpoint inhibitors
NSCLC	Non small cell lung cancer
OS	Overall survival
PFS	Progression-free survival
PET	Positron emission tomography
PLWH	People living with HIV
